# Clinical significance of sarcopenic dysphagia for patients with esophageal cancer undergoing esophagectomy: A review

**DOI:** 10.1002/ags3.12603

**Published:** 2022-07-28

**Authors:** Junya Oguma, Soji Ozawa, Koshiro Ishiyama, Hiroyuki Daiko

**Affiliations:** ^1^ Esophageal Surgery Division National Cancer Center Hospital Tokyo Japan; ^2^ Department of Gastroenterological Surgery, School of Medicine Tokai University Tokyo Japan

**Keywords:** esophageal cancer, rehabilitation, sarcopenic dysphagia, swallowing function

## Abstract

The relationships among esophagectomy for esophageal cancer, dysphagia, and sarcopenia are still unclear. We considered appropriate interventions for patients with resectable esophageal cancer for the purpose of reducing postoperative dysphagia and aspiration pneumonia. Dysphagia in patients with esophageal cancer is caused by patient characteristics, such as pathophysiology and age, or complications after esophagectomy. Recently, sarcopenic dysphagia, defined as dysphagia associated with whole‐body sarcopenia, has attracted attention in various fields, and a large proportion of patients with esophageal cancer are expected to have sarcopenic dysphagia. Our systematic review and meta‐analysis suggested that preoperative sarcopenia in patients with esophageal cancer is related to pulmonary complications after esophagectomy, and some reports also suggested that sarcopenia in swallowing‐related muscles, such as the geniohyoid muscle and tongue, might be associated with postoperative pneumonia or dysphagia after esophagectomy. However, clinical studies on sarcopenic dysphagia in patients with esophageal cancer have been limited. To prevent sarcopenic dysphagia after esophagectomy, perioperative interventions involving not only swallowing rehabilitation, but also physical exercise and nutritional support are important. Moreover, several reports have suggested that the chin‐down maneuver might be effective for preventing aspiration after an esophagectomy. To inhibit the progression of sarcopenic dysphagia after esophagectomy, evaluations and interventions by multidisciplinary staff are likely to be necessary.

## Graphical abstract

Our systematic review and meta‐analysis suggested that preoperative sarcopenia in patients with esophageal cancer is related to pulmonary complications after esophagectomy, and some reports also suggested that sarcopenia in swallowing‐related muscles might be associated with postoperative pneumonia or dysphagia after esophagectomy. to prevent sarcopenic dysphagia after esophagectomy, perioperative interventions involving not only swallowing rehabilitation, but also physical exercise and nutritional support are important.

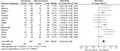

## 1 INTRODUCTION

Recently, minimally invasive esophagectomy and perioperative management have been adopted with the aim of reducing surgical complications after esophagectomy.^1^ However, pulmonary complications after esophagectomy sometimes lead to fatalities^2^; therefore, prevention is an important consideration in perioperative management. One of the most important reasons for postoperative pneumonia is dysphagia. Old age, malnutrition, sarcopenia, multiple primary cancers, vocal code paralysis, and so on have been implicated in postoperative dysphagia, and these characteristics are often features of esophageal cancer patients and the required surgical procedures.

Several previous reports have suggested a relationship between sarcopenia and surgical outcomes after esophagectomy,^3^ and dysphagia caused by sarcopenia has recently become a topic in various fields. However, only a few reports have suggested a relationship between dysphagia arising from sarcopenia and surgical outcomes after esophagectomy, and interventional approaches for preventing dysphagia remain unclear.

The aim of this review was to summarize previous reports suggesting a relationship among esophagectomy for esophageal cancer, dysphagia, and sarcopenia. Appropriate interventions to reduce postoperative dysphagia and aspiration pneumonia in patients with resectable esophageal cancer will also be considered.

## 2 MATERIALS AND METHODS

To evaluate the relationship between preoperative sarcopenia and pulmonary complications after esophagectomy in patients with esophageal cancer, a structured search was conducted using PubMed, the Cochrane Library, and Web of Science. The English search terms were “esophagectomy” AND “sarcopenia” AND “pulmonary complication.” The reference lists of all the included studies were also searched to identify additional studies of possible relevance. The inclusion criteria were a retrospective or prospective cohort study, patients with esophageal or esophagogastric junctional cancer, an evaluation of preoperative sarcopenia, and an evaluation of pulmonary complications, including pneumonia, after esophagectomy. The exclusion criteria were review articles, conference abstracts, non‐English articles, and duplicated publications. The details of the included studies are shown in Table 1. The meta‐analysis was performed using Review Manager 5.3 software.

**TABLE 1:** Summary of studies on the relationship between preoperative sarcopenia and pulmonary complication after esophagectomy in patients with esophageal cancer

Authors	Year	Resion	Study design	Cohort size	Sarcopenia	Evaluation of sarcopenia	PC in patients with sarcopenia	Characteristics of cohort
Ida et al	2015	Japan	Retrospective	138 patients	61 (44%)	BIA	21 patients (34%)	
Makiura D et al	2016	Japan	Retrospective	104 patients	29 (28%)	BIA	11 patients (38%)	
Nishigori T et al	2016	Japan	Retrospective	199 patients	149 (75%)	CT (L3 SMI)	47 patients (32%)	
Elliott JA et al	2017	Ireland	Retrospective	207 patients	49 (24%)	CT (L3 SMI)	27 patients (55%)	Neoadjuvant therapy
Paireder M et al	2017	Austria	Retrospective	130 patients	80 (62%)	CT (L3 SMI)	11 patients (14%)	Neoadjuvant therapy
Jarvinen T et al	2018	Finland	Retrospective	115 patients	92 (80%)	CT (L3 SMI)	26 patients (28%)	Neoadjuvant therapy
Saeki H et al	2018	Japan	Retrospective	157 patients	85 (54%)	CT (L3 SMI)	8 patients (9%)	NACRT
Siegal SR et al	2018	USA	Retrospective	173 patients	127 (73%)	CT (L3 SMI)	18 patients (14%)	
Xu J et al	2019	China	Retrospective	141 patients	73 (52%)	CT (L3 SMI)	52 patients (71%)	MIE
Matsunaga T et al	2019	Japan	Retrospective	163 patients	82 (50%)	BIA	11 patients (13%)	
Oguma J et al	2019	Japan	Retrospective	194 patients	28 (14%)	CT (L3 SMI)	7 patients (25%)	Superficial ESCC
Soma D et al	2019	Japan	Retrospective	102 patients	45 (44%)	CT (L3 SMI)	20 patients (44%)	
Kurita D et al	2020	Japan	Retrospective	161 patients	19 (12%)	HGS	9 patients (47%)	MIE, Male
Srpcic M et al	2020	Slovenia	Retrospective	139 patients	23 (17%)	CT (L3 SMI)	13 patients (57%)	

Abbreviations: BIA, bioelectrical impedance analysis; CT, computed tomography; ESCC, esophageal squamous cell carcinoma; HGS, handgrip strength; MIE, minimally invasive esophagectomy; NACRT, neoadjuvant chemoradiotherapy; PC, pulmonary complication; SMI, skeletal muscle index.

## 3 DYSPHAGIA

### 3.1 Mechanism of dysphagia

Swallowing involves a series of processes: the intake of food into the oral cavity, the formation of a food bolus through chewing, and the passage of the food bolus through the pharynx and esophagus. Dysphagia arises from a disorder in one of these processes. The major symptoms of dysphagia are aspiration and residue. Aspiration means that sputa, food, and drink enter the larynx and trachea. Residue means that the food bolus remains in the oral cavity or pharynx because of a reduction in transfer.^4^

### 3.2 Evaluation of dysphagia

Initially, the repetitive saliva swallowing test (RSST) was used as a screening examination for patients with dysphagia.^5^, ^6^ Later, a videofluoroscopic swallowing test (VFSS) or an endoscopic evaluation of swallowing also began to be performed, as required. The movement of the vocal cords, the response of the larynx, the pharyngeal residue of saliva, and its penetration into the larynx are typically evaluated using an endoscopic examination,^7^ and the Hyodo–Komagome score is usually used as an endoscopic scoring system in Japan.^8^ On the other hand, the degree of penetration into the larynx and trachea and their response can be measured using VFSS according to a penetration‐aspiration scale (PAS).^9^, ^10^, ^11^

Transportation of the bolus during swallowing can be observed, and dysphagia and pharyngeal residue can be evaluated using both examinations. Additionally, the range, speed, and timing of various organ movements associated with swallowing can also be analyzed, and the mechanisms of swallowing and pharyngeal residue can be investigated. If these mechanisms are intact, rehabilitation to solve the swallowing problems is possible.

### 3.3 Relationship between dysphagia and esophageal cancer

There are various reasons for dysphagia after esophagectomy for patients with esophageal cancer. These reasons are shown below.

#### 3.3.1 Pathophysiology

Many patients with advanced esophageal cancer have dysphagia before treatment because of severe stenosis caused by a large primary tumor or swelling of the metastatic lymph nodes along the esophagus. Patients with malnutrition can also have poor swallowing. Reportedly, 15%–30% of esophageal cancer patients have multiple primary cancers, and the rate of head and neck cancer was particularly high.^12^, ^13^ Esophageal cancer patients with a history of surgery or radiotherapy in the head and neck region often have poor swallowing before treatment for esophageal cancer.^14^, ^15^

#### 3.3.2 Aging

Most esophageal cancer patients are elderly; for example, most patients with esophageal cancer in Japan are in their 60s or 70s.^16^ Therefore, a reduction in swallowing because of aging is often considered to be an underlying cause. Presbyphagia is not a disease; however, a slight change in swallowing function, such as delayed transportation in the oral cavity, a reduction in pharyngeal muscle strength, or an increase in pharyngeal residue, was found in elderly patients, and penetration into the larynx and aspiration can occur in such patients.^17^, ^18^

#### 3.3.3 Dysphagia after esophagectomy

The following things are considered to be causes of dysphagia: an abnormal larynx elevation caused by scarring around the trachea and larynx, a reduced cough reflux caused by a decrease in blood flow in the trachea, the bending of reconstructed organs, and a decrease in the swallowing pressure caused by laryngeal nerve paralysis. An elevated larynx can cause obstruction because the isolation of the infrahyoid muscle during cervical lymph node dissection can prevent it from relaxing during subsequent swallowing.^19^, ^20^, ^21^ This can induce an obstruction in the upper esophagus and a closing insufficiency of the larynx, leading to a decrease in pharynx clearance and penetration into the larynx. Laryngeal nerve paralysis can be reduced with a surgeon's effort, because this is a postoperative complication caused by the surgical procedure. On the other hand, scarring around the trachea and a decrease in tracheal blood flow are unavoidable, as they are normal conditions after surgery.

In terms of the relationship between surgical procedure and postoperative dysphagia, a three‐field lymphadenectomy, compared with a two‐field lymphadenectomy,^19^ and retrosternal reconstruction^20^ were reportedly related to dysphagia.

## 4 SARCOPENIC DYSPHAGIA

### 4.1 Relationship between sarcopenia and outcomes after esophagectomy

**FIGURE 1:**
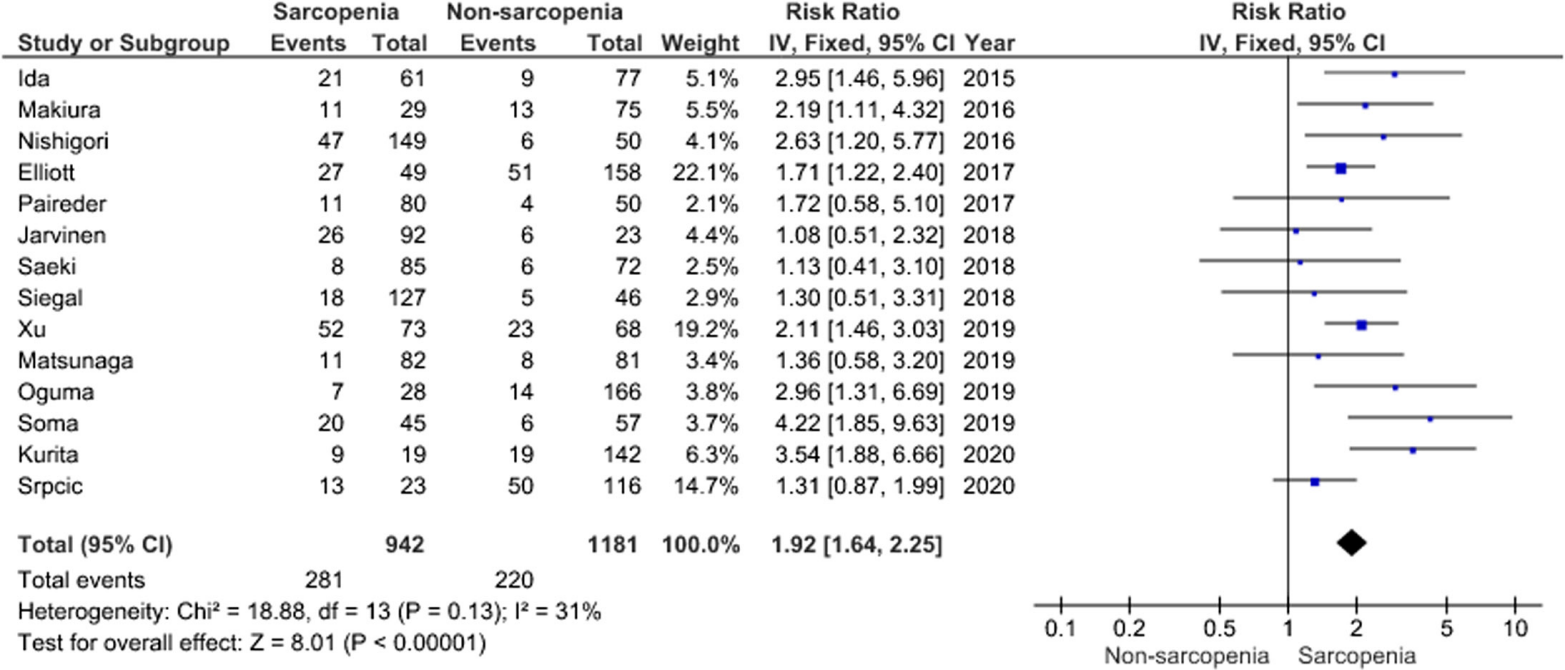
A meta‐analysis of pulmonary complications after esophagectomy in patients with sarcopenia and esophageal cancer

The concept of sarcopenia was first introduced in 1989 as an age‐dependent decline in muscle mass, strength, and physical function.^22^ The diagnostic criteria for sarcopenia were revised in 2019, and sarcopenia was defined as a gradual and generalized loss of skeletal muscle strength and mass. Moreover, severe sarcopenia was diagnosed as the additional loss of physical condition. Sarcopenia can derive from not only aging, but also systemic disease such as malnutrition, advanced organ failure, inflammatory disease, and malignancy.^23^, ^24^ Several patients with esophageal cancer are likely to be diagnosed as having sarcopenia, since many are elderly and have malnutrition or dysphagia.^25^ Several reports have shown that preoperative sarcopenia is associated with postoperative pulmonary complications (PC) among patients undergoing surgery for esophageal cancer.^26^, ^27^, ^28^ On the other hand, some reports have suggested that sarcopenia is not associated with PC after esophagectomy.^29^, ^30^ To assess the relationship between preoperative sarcopenia and PC after esophagectomy in previously reported patients with esophageal cancer, a structured review was conducted, as mentioned previously. Finally, a total of 14 references were included in this review^27^, ^28^, ^29^, ^30^, ^31^, ^32^, ^33^, ^34^, ^35^, ^36^, ^37^, ^38^, ^39^, ^42^ (Table 1). Among the included studies, the prevalence of preoperative sarcopenia ranged from 12%–80%, while the prevalence of PC after esophagectomy ranged from 9%–71%. A meta‐analysis of the included studies revealed that preoperative sarcopenia significantly increased the risk of pulmonary complications after esophagectomy in patients with esophageal cancer (risk ratio = 1.92, 95% confidence interval [CI] = 1.64, 2.25, *P* < .00001) (Figure 1). However, a relationship between swallowing function and PC was not reported in any of the included studies. In the future, the possible association of sarcopenic dysphagia with PC after esophagectomy in patients with esophageal cancer should be evaluated.

Additionally, some reports suggested that sarcopenia is also associated with a poor prognosis after surgery.^40^, ^41^, ^42^ Moreover, several reports have suggested that sarcopenia in patients with locally advanced esophageal cancer is associated with the morbidity of dose‐limiting toxicities after neoadjuvant chemotherapy or chemoradiotherapy.^43^, ^44^ A loss of skeletal muscle mass during neoadjuvant therapy has also been reported to be associated with postoperative complications^45^ and long‐term survival.^46^

### 4.2 Definition of sarcopenic dysphagia

In 2012, Kuroda et al first reported a correlation between arm muscle mass and swallowing function,^47^ and sarcopenia was subsequently suggested to be an independent risk factor of dysphagia.^48^, ^49^ A diagnostic algorithm for dysphagia associated with sarcopenia was created by the Working Group on Sarcopenic Dysphagia in Japan in 2017. Dysphagia caused by whole‐body sarcopenia was first categorized as “sarcopenic dysphagia” in 2019.^50^

Swallowing‐related muscles, such as the tongue, the geniohyoid muscle, and the pharyngeal wall were evaluated using an ultrasound study,^51^ a computed tomography study,^52^ and a magnetic resonance imaging study,^53^ and these muscle masses were found to be related to aging.^49^ The geniohyoid muscle mass was also related to tongue pressure and jaw‐opening strength,^54^ and tongue pressure was related to dysphagia in older individuals.^55^, ^56^ Because of the difficulty in measuring swallowing‐related muscle volumes, the diagnostic algorithm for sarcopenic dysphagia, mentioned above, was developed to include only muscle strength.

### 4.3 Sarcopenic dysphagia and esophageal cancer

Clinical studies on sarcopenic dysphagia in patients with esophageal cancer have been limited until now. Only three previous reports have evaluated swallowing function or dysphagia in patients undergoing esophagectomy. Mayanagi et al suggested that preoperative sarcopenia and laryngeal nerve palsy were independent risk factors of postoperative dysphagia in 187 patients with esophageal cancer in a retrospective study.^25^ Katsumata et al reported that a reduction in geniohyoid muscle mass caused dysphagia in patients after surgery for esophageal cancer^57^; furthermore, Yokoi et al suggested that a decrease in tongue pressure before and after surgery was significantly associated with postoperative pneumonia among inpatients with esophageal cancer after esophagectomy^58^ (Table 2). These findings suggested that dysphagia after esophagectomy seemed to be related to factors associated with patient characteristics; therefore, perioperative interventions for patients with esophageal cancer might improve their swallowing function. Recently, neoadjuvant therapy has become the gold standard for patients with locally advanced esophageal cancer. Therefore, the relationship between swallowing function and postoperative outcomes should be evaluated, taking into consideration the influence of neoadjuvant therapy.

**TABLE 2:** Relationship between sarcopenia and dysphagia in patients with esophageal cancer

References	Yokoi et al	Katsumata et al	Mayanagi et al
Reported date	2019	2019	2021
Study design	Longitudinal study	Retrospective study	Retrospective study
Sample size	59	54	187
Measurement	Tongue pressure measurement, RSST	Geniohyoid muscle mass (CT image), VF	PMI (CT image), VF, FEES
Evaluation	Change in tongue pressure	Change in geniohyoid muscle mass	Sarcopenia as decrease of PMI
Outcomes	Decrease in tongue pressure was associated with the length of ICU stay.	Decrease in geniohyoid muscle mass causes the dysphagia.	Sarcopenic patients with esophageal cancer developed postoperative dysphagia.

Abbreviations: FEES, fiberoptic endoscopic evaluation of swallowing; ICU, intensive care unit; PMI, psoas muscle mass index; RSST, repetitive saliva swallowing test; VF, videofluoroscopic swallowing study.

## 5 INTERVENTIONS FOR SARCOPENIC DYSPHAGIA

### 5.1 Preoperative interventions

A few previous reports have suggested evidence for rehabilitative interventions for sarcopenic dysphagia. Wakabayashi et al reported a randomized control trial evaluating swallowing‐related muscle training using a tongue resistance exercise and a head flexion exercise for 91 elderly patients with dysphagia; the results suggested that resistance training of the swallowing muscles did not improve dysphagia.^59^ On the other hand, Maeda et al reported a case with sarcopenic dysphagia in which comprehensive care, including aggressive nutritional support and rehabilitation, resulted in improvement.^60^ These findings suggest that interventions for sarcopenic dysphagia seem to require both dysphagia rehabilitation and nutritional support.

Several patients with esophageal cancer are elderly; therefore, many of them have malnutrition or sarcopenia before treatment. The possible presence of sarcopenia or dysphagia should be evaluated in these patients by dentists, physical therapists, speech therapists, and nutritionists at the time when the treatment strategy is decided. When dysphagia is diagnosed in patients, the causes of the dysphagia should be clarified. Most patients with locally advanced esophageal cancer receive neoadjuvant therapy for several months; therefore, rehabilitative and nutritional interventions by each specialist are possible during neoadjuvant therapy.

### 5.2 Postoperative interventions

Generally, physical training is started on postoperative day 1 after an esophagectomy; however, the starting time and details of swallowing training after an esophagectomy are still controversial, and there is no evidence showing a clinical effect of postoperative dysphagia intervention. Considering the load of anastomosis and aspiration, indirect training for swallowing is usually undergone until the beginning of oral ingestion; thereafter, direct training is performed. As mentioned above, the swallowing function is decreased by an esophagectomy; therefore, continuous swallowing training before and after surgery and improvement of the swallowing procedure are important. Okumura et al suggested that perioperative swallowing rehabilitation, including pursed lip breathing, a cervical range of motion exercise, shoulder stretches, jaw opening, tongue exercises, and submental muscle training did not change swallowing biomechanics but decreased the volume of laryngeal and pharyngeal residue in patients after an esophagectomy.^61^ The chin‐tuck maneuver has been recommended as a swallowing method after an esophagectomy in many reports. This maneuver was suggested to improve airway protection and pyriform sinus residue and to increase the upper esophageal sphincter (UES) opening diameter and prolong the duration of UES opening, compared with the neural position^62^, ^63^, ^64^ (Table 3).

**TABLE 3:** Rehabilitative intervention for patients with esophageal cancer after esophagectomy

References	Lewin et al	Okumura et al	Kumai et al	Kumai et al
Reported date	2001	2016	2017	2017
Study design	Retrospective study	Retrospective study	Retrospective study	Prospective study
Sample size	26	14	25	14
Interventions	Chin‐tuck maneuver during swallowing	Swallowing exercise 1. Pursed‐lip breathing 2. Cervical range of motion exercise 3. Shoulder stretch 4. Jaw opening 5. Tongue exercises	Chin‐down maneuver combined with supraglottic swallow (CDSS)	Chin‐down maneuver
Evaluation	VF	VF	VF	VF
Conclusions	Chin‐tuck maneuver protects the airway of patients following esophagectomy.	Swallowing function after esophagectomy was improved by perioperative SR.	Laryngeal aspiration following esophagectomy can be ameliorated after training in the CDSS.	Chin‐down maneuver after esophagectomy can help expedite swallowing.

Abbreviations: SR, swallowing rehabilitation; VF, videofluoroscopic swallowing study.

## 6 CONCLUSION

The causes of dysphagia after esophagectomy are numerous, and previous reports have suggested that these factors are closely associated with sarcopenic dysphagia. There has been no evidence regarding interventions for esophageal cancer patients with sarcopenic dysphagia for the purpose of preventing postoperative dysphagia until now; therefore, further evaluations are expected in the future. Importantly, sarcopenia and dysphagia should be accurately evaluated before surgery, and interventions should be consistently performed both before and after esophagectomy. Not only swallowing interventions for dysphagia, but also physical therapy and nutritional support for sarcopenia should be performed simultaneously in cooperation with a multidisciplinary staff. Moreover, a guarantee of manpower resources to provide the intervention and the adherence of patients continuing the intervention are also extremely important. To solve these problems, interventional manuals for medical staff and brochures for patients should be created to allow them to understand fully the significance of interventions and to enhance the motivation of patients.

Because of the development of multimodal therapy for esophageal cancer, which is a refractory cancer, treatment outcomes have been improved. In the future, medical staff should emphasize not only the “cure,” but also the “care” of patients with esophageal cancer after an esophagectomy. To do this, evaluations and interventions by a multidisciplinary staff who are aware of sarcopenic dysphagia will be important.

## DISCLOSURE

Funding: No funding was received for this study.

Conflict of Interest: The authors have no conflicts of interest to declare.

Author Contributions: Junya Oguma: literature search, study design, analysis plan, data analysis, and interpretation, drafting of article. Soji Ozawa: study design, data interpretation and analysis, article revision. Koshiro Ishiyama: study design, data interpretation and analysis. Hiroyuki Daiko: study design, data interpretation and analysis, article revision.

## ETHICAL APPROVAL

The protocol for this research project has been approved by a suitably constituted Ethics Committee of the institution and it conforms to the provisions of the Declaration of Helsinki. The Ethics Committee of the National Cancer Center Hospital, Approval No. 2020‐287.
